# Influence of acquisition settings and radiation exposure on CT lung densitometry—An anthropomorphic *ex vivo* phantom study

**DOI:** 10.1371/journal.pone.0237434

**Published:** 2020-08-14

**Authors:** Patricia Leutz-Schmidt, Mark O. Wielpütz, Stephan Skornitzke, Oliver Weinheimer, Hans-Ulrich Kauczor, Michael U. Puderbach, Gregor Pahn, Wolfram Stiller

**Affiliations:** 1 Diagnostic and Interventional Radiology (DIR), Heidelberg University Hospital, Heidelberg, Germany; 2 Translational Lung Research Center (TLRC) Heidelberg, German Center for Lung Research (DZL), University of Heidelberg, Heidelberg, Germany; 3 Department of Diagnostic and Interventional Radiology with Nuclear Medicine, Thoraxklinik at Heidelberg University Hospital, Heidelberg, Germany; 4 Department of Radiology (E010), German Cancer Research Center (DKFZ), Heidelberg, Germany; INSERM, FRANCE

## Abstract

**Objectives:**

To systematically evaluate the influence of acquisition settings in conjunction with raw-data based iterative image reconstruction (IR) on lung densitometry based on multi-row detector computed tomography (CT) in an anthropomorphic chest phantom.

**Materials and methods:**

Ten porcine heart-lung explants were mounted in an *ex vivo* chest phantom shell, six with highly and four with low attenuating chest wall. CT (Somatom Definition Flash, Siemens Healthineers) was performed at 120kV_p_ and 80kV_p_, each combined with current-time products of 120, 60, 30, and 12mAs, and was reconstructed with filtered back projection (FBP) and IR (Safire, Siemens Healthineers). Mean lung density (LD), air density (AD) and noise were measured by semi-automated region-of interest (ROI) analysis, with 120kV_p_/120 mAs serving as the standard of reference.

**Results:**

Using IR, noise in lung parenchyma was reduced by ~ 31% at high attenuating chest wall and by ~ 22% at low attenuating chest wall compared to FBP, respectively (p<0.05). IR induced changes in the order of ±1 HU to mean absolute LD and AD compared to corresponding FBP reconstructions which were statistically significant (p<0.05).

**Conclusions:**

Densitometry is influenced by acquisition parameters and reconstruction algorithms to a degree that may be clinically negligible. However, in longitudinal studies and clinical research identical protocols and potentially other measures for calibration may be required.

## Introduction

Quantitative post-processing of computed tomography (CT) image data based on measurements of lung attenuation in Hounsfield units (HU) has become a clinically accepted tool to assess emphysema and air-trapping in chronic obstructive pulmonary disease (COPD) in clinical practice and research [1–5]. Against this background, in reaction to concerns regarding risks from cumulating patients’ radiation exposure with repeated examinations, low-dose computed tomography has been introduced to limit radiation exposure. In general, exposure reduction in CT is hindered by the increase in image noise which is associated with degraded image quality and diagnostic performance [6]. Aside of the conventional filtered back projection (FBP), that has been the standard reconstruction algorithms for years, iterative reconstruction (IR) [7] and even deep-learning based reconstruction algorithms have recently been introduced, with the latter already being available for clinical use from two major CT manufacturers to address this obstacle [8]. Recent data using abdominal phantoms show that IR, using statistical models for image noise reduction, may maintain diagnostic image quality at a reduction of radiation dose by up to 50% [9–12].

In a recent *ex vivo* phantom study, we could demonstrate that quantitative airway parameters such as wall thickness are not affected by exposure reduction or IR in a meaningful manner [13]. At present, however, the influence of IR on lung densitometry is largely unknown [4], while it is specifically known that CT exposure reduction does not only affect noise but also density of examined materials such as lung tissue [14–16]. Therefore, the aim of our current investigation was to evaluate the influence of acquisition settings in conjunction with raw data-based IR on lung densitometry with semi-automatic analysis in a previously evaluated anthropomorphic *ex vivo* porcine chest phantom, which allows for an arbitrary number of CT acquisitions without requiring any radiation exposure to patients.

## Materials and methods

### Anthropomorphic *ex vivo* porcine chest phantom

**Fig 1:**
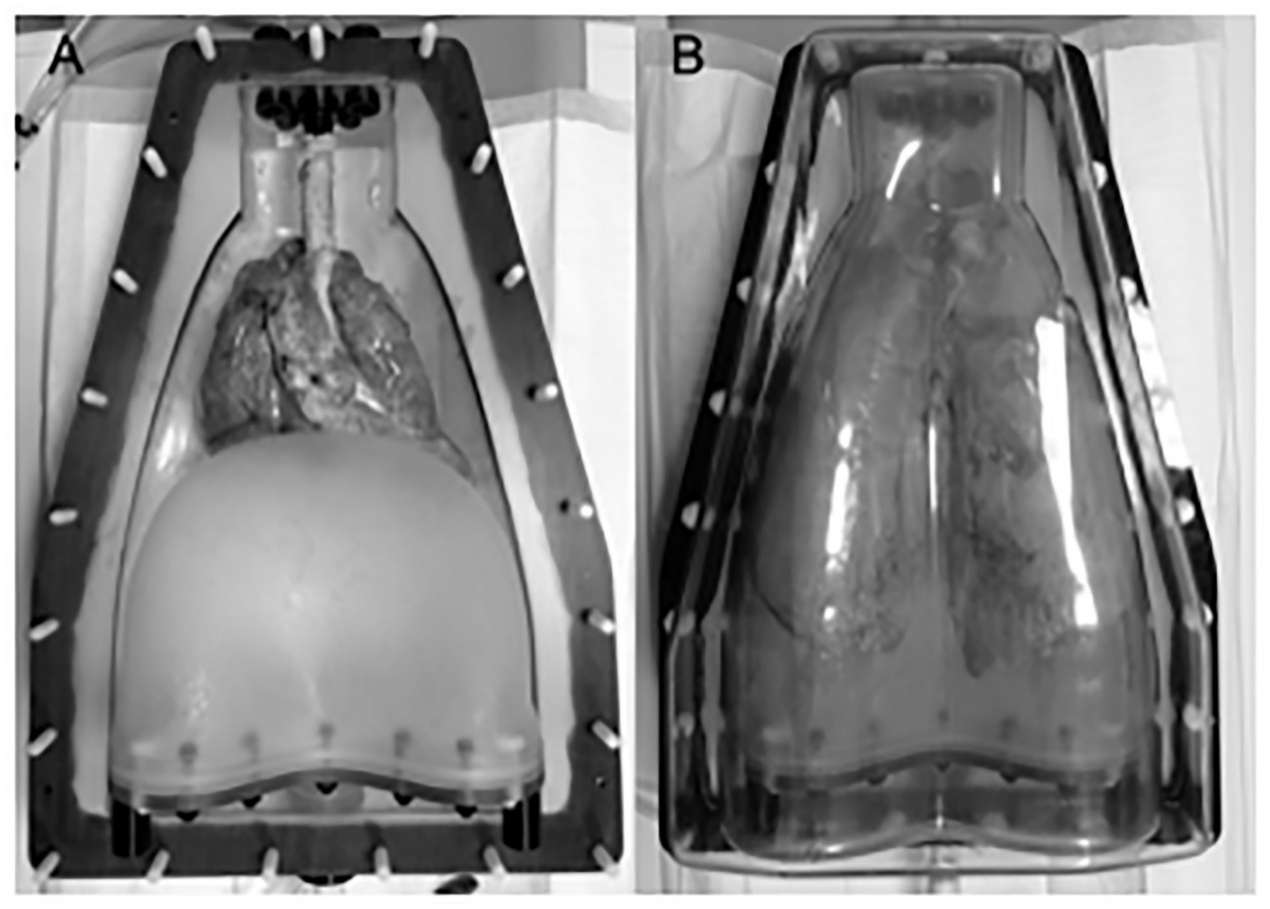
Anthropomorphic *ex vivo* porcine chest phantom. (A) The porcine heart-lung explant in the artificial phantom shell (Artichest, PROdesign GmbH, Heiligkreuzsteinach, Germany), simulating the attenuating thoracic anatomy surrounding the lungs inflated with ambient air. (B) After mounting of the second half of the shell, constant negative pressure was applied to the “pleural space” to keep lungs inflated and the phantom was flipped to “supine”. Multi-row detector computed tomography was performed immediately after successful phantom setup.

No animal was harmed for the purpose of this research. Ten domestic porcine heart-lung explants (without the thoracal sceleton) were obtained from a local abattoir in Mannheim, Germany (Fleischversorgungszentrum Mannheim, Schlachthofstr.21, 68165 Mannheim). Great care was taken not to damage the surface of the lung explants. For image acquisition, the ten freshly excised porcine heart-lung explants were used together with a commercially available lung phantom apparatus. This system uses a copolymer container constructed to simulate a chest that holds the heart and inflated lung explant of a pig by continuous evacuation of the artificial pleural space with 2–3 × 10^3^ Pa (Fig 1). The shell as well as a rubber dome can be filled with pure water to simulate the attenuation of the chest wall and upper abdomen, respectively, (Artichest, PROdesign GmbH, Heiligkreuzsteinach, Germany) which has been described previously [13, 17–21].

Of the ten explants, four lungs were used for simulating a high attenuating chest wall (water-filled phantom container and artificial diaphragm), and the remaining six lungs for simulating a low attenuating chest wall (air-filled phantom container and artificial diaphragm). The apparatus is capable of simulating respiratory motion, and a fixed breathhold position at full inflation was selected for this study.

### Multi-row detector computed tomography

Image datasets were acquired in spiral (helical, pitch factor 0.9) technique using a regularly calibrated 64-detector row 128 slice CT system (Somatom Definition Flash, Siemens Healthineers, Forchheim, Germany). Each dataset acquired was reconstructed with both FBP (B40f kernel, medium-soft) and corresponding IR (I40f kernel, medium-soft) at 0.75 mm slice thickness, 128x0.6 mm collimation, 0.6 mm increment, 512x512 pixel matrix, and 300×300 mm^2^ field-of-view. The medium-soft kernel was chosen as it is recommended for use in densitometry [19, 20, 22, 23]. For IR, a raw-data based algorithm (Safire, Siemens Healthineers, Forchheim, Germany) was used and “strength” 3 of 5 was selected as previously described [13, 19, 20, 24]. With ten lung explants, eight dose levels, and two reconstructions per acquisition, 160 datasets were obtained for this comparative study. Exposure settings were chosen to represent “moderate” to “low-dose” chest CT protocols as currently used in clinical routine, and to challenge IR by acquisitions at very low dose (Table 1). Of note, automatic exposure control (AEC) was disabled to ensure homogenous exposure of the whole scanning volume. Image data can be made available by the authors upon request.

**Table 1:** Influence of acquisition parameters, radiation exposure, and image reconstruction algorithm on mean lung and air density and image noise measured in an anthropomorphic *ex vivo* chest phantom simulating low attenuation chest wall (air-filled phantom container and artificial diaphragm).

Algorithm	Tube potential [kV_p_]	Current-time product [mAs]	CTDI_vol_ [mGy]	Lung density [HU]	Noise in lungs [HU]	Air density [HU]	Noise in air [HU]
**FBP**	**120**	**120**	8.1	-920.5±6.6*#	16.0±2.0*	-998.8±1.1*	5.5±0.4
		**60**	4.1	-920.3±5.9*	17.0±2.0	-999.1±1.1	7.2±0.6
		**30**	2.0	-920.4±6.2*	18.1±2.2	-999.7±1.0	9.1±0.4
		**12**	0.9	-920.0±6.2*	20.7±2.0*	-999.5±0.7	12.9±0.4*
	**80**	**120**	2.4	-923.4±6.4*	17.2±1.8	-999.3±1.2	8.5±0.5
		**60**	1.2	-923.7±6.4	18.9±2.1*	-999.8±1.0	11.1±0.6*
		**30**	0.6	-928.0±6.3	21.9±2.1*	-998.5±1.1*	14.6±0.6*
		**12**	0.3	-926.6±6.4	27.6±2.1*	-994.7±0.9*	20.4±0.5*
**IR**	**120**	**120**	8.1	-920.4 ±6.6*#	14.3±2.1	-998.3±1.1	4.2±0.5
		**60**	4.1	-920.3±5.9*	15.0±2.1	-998.6±1.1	5.3±0.7
		**30**	2.0	-920.2±6.2*	15.7±2.2	-999.0±1.0	6.6±0.5*
		**12**	0.9	-918.5±6.1*	20.6±5.4*	-996.6±4.1	12.5±6.1*
	**80**	**120**	2.4	-923.2±6.4	15.1±1.9	-998.7±1.2	6.3±0.5
		**60**	1.2	-923.3±6.4	16.1±2.0	-999.1±1.0	8.1±0.6*
		**30**	0.6	-927.3±6.2	18.0±2.1*	-998.0±1.1*	10.7±0.5*
		**12**	0.3	-925.4±6.3*	21.6±2.1*	-995.3±1.0*	15.1±0.5*

Data given as mean ± SD.

*p<0.05 vs. other exposure parameters within the same reconstruction regime, i.e. “FBP” or “IR”.

^#^All results for FBP differ significantly (p<0.05) from IR except for mean lung density at 120 kV_p_, 120 mAs and for noise (SD) in lungs and air at 120 kV_p_, 12 mAs.

### Quantitative post-processing

**Fig 2:**
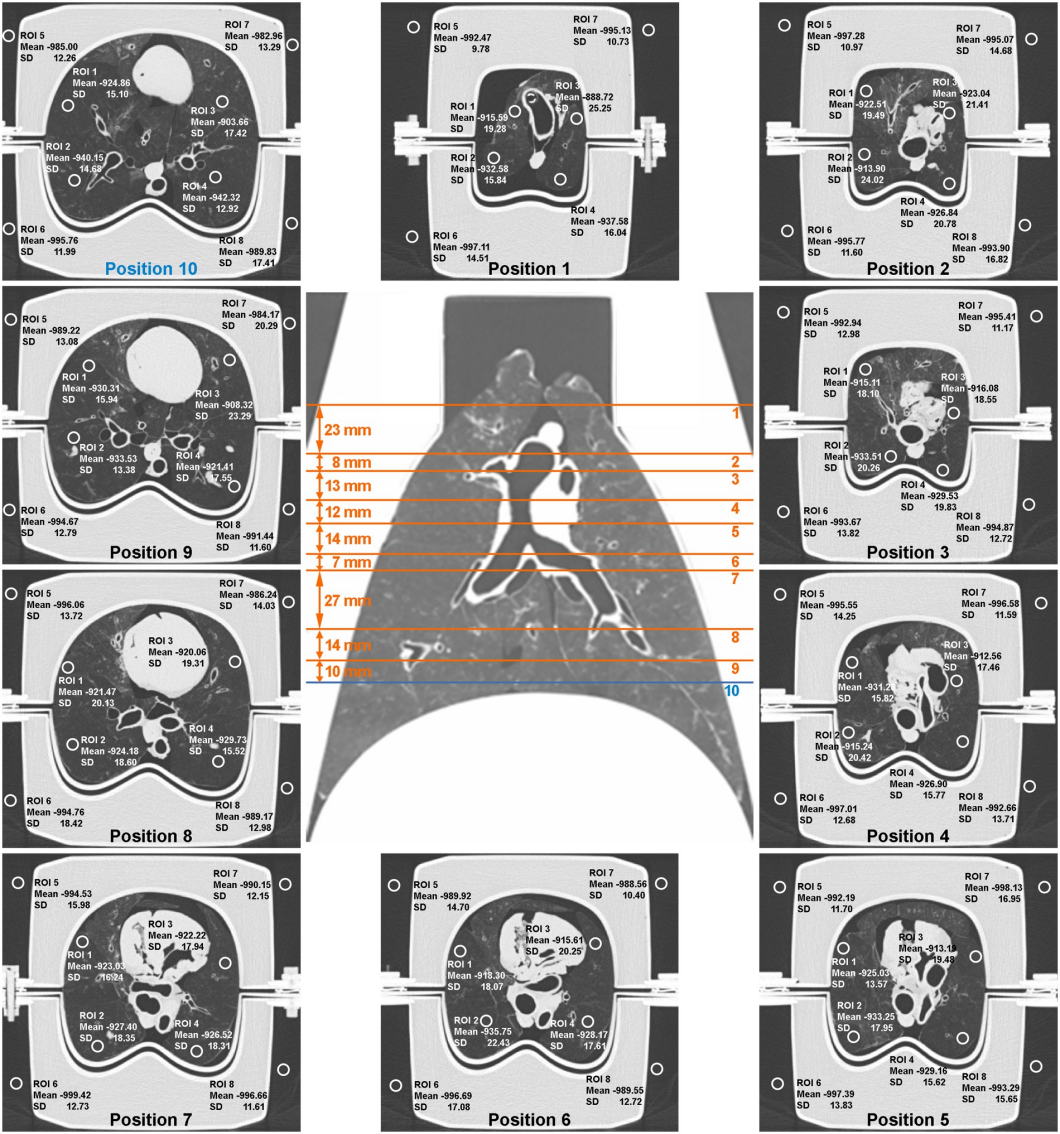
Example of a CT reference acquisition (120 kV_p_, 120 mAs) of the anthropomorphic *ex vivo* porcine chest phantom with high attenuation chest wall. The ten image slices from apical to basal lung selected for region-of-interest (ROI) based evaluation of mean lung (LD) and air density (AD) as well as noise per image dataset are shown and respective readout slice locations are indicated in the coronal reformation along with corresponding inter-readout slice intervals. Note that readout slice location no. 10 (blue) indicates slice location of the image example displayed in Fig 3. All numbers pertaining to a respective ROI are stated in Hounsfield units [HU]. Note the small pneumothorax encompassing parts of the porcine lung. The image slices were selected due to distance to tracheal bifurcation and heart structures.

For each of the ten heart-lung phantom preparations, ten image slices covering the lung from apex to base and located at intervals from ~10 to ~30 mm while aiming for the best possible approximation of even readout slice distribution were selected in the 120 kV_p_, 120 mAs acquisition as the reference image dataset (Fig 2). Matching of these reference readout slice locations between each of the ten heart-lung phantom preparations was aimed for by careful selection of matching slices based on anatomical landmarks in each of the ten reference image datasets.

**Fig 3:**
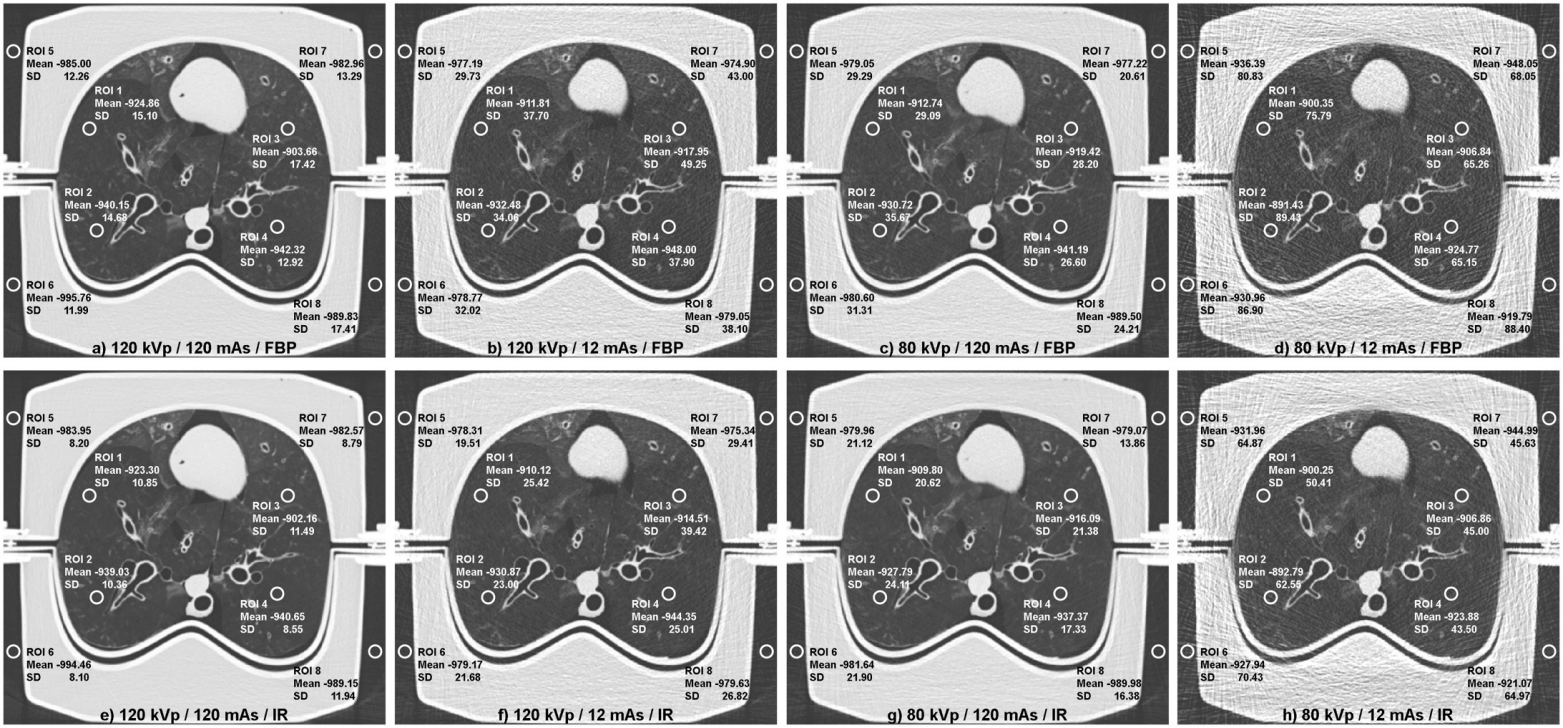
Region-of-interest (ROI) placement for lung and air density measurement. Four circular ROIs of ~25 mm^2^ were placed each in lung parenchyma and in extracorporeal air for measurements of lung density (LD) and air density (AD). Filtered back projection reconstructions are displayed in the upper row, iterative reconstructions within the bottom row. For each of the ten heart-lung phantom preparations, ROI placement encompassed ten image slices at locations from apical to basal lung (cf. Fig 2). Image slices shown correspond to location number ten indicated in blue color in the coronal lung surview of Fig 2C. Note that all numbers pertaining to a respective ROI are stated in Hounsfield units [HU].

On each readout slice of the reference image datasets, a radiologist placed four circular regions-of-interest (ROIs) of equal size (i.e. covering an area of ~25 mm^2^) in the inflated lung parenchyma excluding major vessel and airways, together with an additional four ROIs (same shape and size) in the air surrounding the phantom (Figs 2 and 3), resulting 80 equally sized (~25 mm^2^) circular ROIs per acquired image dataset, i.e. eight ROI per image slice for each of the ten image slices from apical to basal lung selected per image dataset.

As each heart-lung phantom preparation was imaged and reconstructed in exactly the same position and with identical FOV, the resulting 80 equally sized (~25 mm^2^) circular ROIs were automatically propagated from the reference image dataset to each of the image datasets originating from the other acquisitions and reconstructions of the same heart-lung phantom preparation, thus allowing for quantitative evaluation within standardized ROI. All of the aforementioned steps were carried out for each of the ten heart-lung phantom preparations. Subsequently, the ROIs were evaluated quantitatively in terms of mean HU for lung density (LD) and for air density (AD), respectively, and for noise in lung and air (standard deviation of HU) using an image analysis software developed in-house described previously [25]. For obtaining mean LD and corresponding noise in the lungs, the CT numbers or their respective standard deviation measured in all ROIs placed in inflated lung parenchyma (excluding major vessel and airways) were averaged across all ten image slices; the same was done for obtaining mean AD and noise in air by using all ROIs placed in the air surrounding the phantom. The aforementioned software used for ROI analysis in the context of this study is a research software framework for quantitative assessment of image quality (IQ) in CT developed in C++ entirely based on open-source software libraries for medical image processing. The framework has been designed in a modular and object-oriented fashion, separating algorithms (e.g. for the determination of IQ quantities) from classes for handling geometrical objects (e.g. image data, phantoms, ROIs), thus facilitating its effortless extension and flexible customization e.g. by inclusion of other calculation methods and adaptation to different phantom geometries. Thus, the software enables systematic, reproducible, automated and time-efficient quantitative IQ analysis while also offering comprehensive customizable graphical presentation, analysis, and data export of all results with full user control [24].

### Statistical analysis

Analysis was done with SigmaPlot software (Systat Software GmbH, Erkrath, Germany). For comparison of different acquisition settings within the groups “FBP” and “IR” repeated measures analysis of variance (RM ANOVA on ranks), and for comparison of “FBP” vs. “IR” at identical acquisition settings Wilcoxon signed rank test was used. Similarly, high and low attenuation chest wall were compared. Significance levels were adjusted for multiple comparison as appropriate, and a p-value < 0.05 was considered statistically significant [26].

## Results

### Influence of acquisition settings on noise

Mean noise in lung was significantly higher in the phantom mimicking high attenuation chest wall compared to low attenuation chest wall (p<0.001) at all acquisition settings with FBP and IR, except for 120 kV_p_ 120 mAs (n.s.) (Tables 1 and 2).

**Table 2:** Influence of acquisition parameters, radiation exposure, and image reconstruction algorithm on mean lung and air density and image noise measured in an anthropomorphic *ex vivo* chest phantom simulating high attenuating chest wall (water-filled phantom container and artificial diaphragm).

Algorithm	Tube potential [kV_p_]	Current-time product [mAs]	CTDI_vol_ [mGy]	Lung density [HU]	Noise in lungs [HU]	Air density [HU]	Noise in air [HU]
**FBP**	**120**	**120**	8.1	-917.3±8.1	17.9±2.4	-995.1±3.1	12.8±1.8
		**60**	4.1	-916.6±7.6	24.9±2.3	-994.3±3.3	17.4±0.9
		**30**	2.0	-917.9±8.1	30.1±5.3	-991.3±3.2	22.1±3.0
		**12**	0.9	-917.3±8.8	45.2±3.5*	-983.2±3.3*	32.1±1.8*
	**80**	**120**	2.4	-917.0±7.6	30.8±2.5*	-988.9±4.1*	24.2±1.2
		**60**	1.2	-917.2±8.6	41.8±3.1	-982.9±3.6*	32.2±1.8*
		**30**	0.6	-916.0±10.2	56.6±3.8*	-971.3±3.1*	45.4±2.2*
		**12**	0.3	-902.5±11.0*	77.0±4.7*	-948.5±5.4*	69.5±3.2*
**IR**	**120**	**120**	8.1	-915.7±7.6	13.8±1.3	-994.7±3.2	9.9±1.0
		**60**	4.1	-915.3±7.5	18.2±2.1	-994.2±3.3	12.3±0.7
		**30**	2.0	-916.9±8.0	23.8±3.3	-991.9±2.5	17.2±2.4*
		**12**	0.9	-916.1±9.0	30.8±2.9*	-984.6±3.3*	21.8±1.0*
	**80**	**120**	2.4	-914.9±8.5	23.5±6.2	-988.8±5.1*	17.9±2.3*
		**60**	1.2	-915.6±8.3	28.5±2.3*	-984.1±3.7*	22.6±1.1*
		**30**	0.6	-914.6±9.8	38.1±2.7*	-970.9±3.4*	31.7±1.3*
		**12**	0.3	-901.6±10.9*	52.7±3.6*	-946.6±5.7*	49.9±2.6*

Data given as mean ± SD.

* p<0.05 vs. other exposure parameters within the same reconstruction regime, i.e. “FBP” or “IR”. All results of FBP differ significantly (p<0.05) from IR except for air density at 120 kV_p_, 60 mAs.

Mean noise in air was significantly higher in the phantom with high attenuation chest wall for all acquisition parameters with FBP and IR (p<0.001).

Irrespective of image reconstruction algorithm used, average noise in lung and noise in air significantly increased with reduced radiation dose, e.g. for FBP noise in lung ranged from 16.0 HU at 120 kV_p_ 120 mAs to 27.6 HU at 80 kV_p_ 12 mAs (+172.5%), and for noise in air from 5.5 HU at 120 kVp to 20.4 HU at 80 kV_p_ 12 mAs (+370.9%) with low attenuation chest wall (Table 1). For high attenuation chest wall the impact of acquisition settings on noise was higher, with a range of noise in lung from 17.9 HU at 120 kV_p_ 120 mAs to 77.0 HU at 80 kV_p_ 12 mAs (+430.2%), and for noise in air from 12.8 HU at 120 kV_p_ 120 mAs to 69.5 HU at 80 kV_p_ 12 mAs (+543.0%) (Table 2).

### Influence of acquisition settings on densitometry

Mean LD and AD were significantly higher in the high attenuation chest wall compared to the low attenuation chest wall phantom at each acquisition setting, for FBP and for IR alike (p<0.001) (Tables 1 and 2). For example, LD was -917.3 HU with the high attenuation chest wall and -920.5 HU (-0.3%) with the low attenuation chest wall phantom in the reference series with FBP. Similarly, for AD it was -995.1 HU and -998.8 HU (-0.4%), respectively.

LD increased with lower radiation dose and ranged from -917.3 HU in the reference series to -902.5 HU (+1.7%) at lowest radiation dose (80 kV_p_ 12 mAs) in the high attenuation chest wall phantom for FBP (p<0.05). Similarly AD ranged from -995.1 HU to -948.5 HU (FBP, p<0.05) (+4.7%). In the low attenuation chest wall phantom only LD decreased with lower radiation dose and ranged from -920.5 HU in the reference series to -926.6 HU at 80 kV_p_ 12 mAs using FBP (p<0.05) (-0.7%). However, AD increased slightly and ranged from -998.8 HU to -994.7 HU (p<0.05) (+0.4%). Comparing low to high attenuation chest wall showed significant differences for AD (p<0.001), except at 120 kV_p_,120mAs (p<0.02).

### Influence of iterative reconstruction on noise

Using IR (SAFIRE) and medium-soft kernel (I40F), on average noise in lung was reduced compared to FBP (B40f) by approx. 31% in the high attenuation chest wall and by approx. 22% in the low attenuation chest wall phantom at the same exposure settings (p<0.001). Similarly, noise in air was reduced by approx. 28% in the high attenuation chest wall and approx. 26% in the low attenuation chest wall phantom (p<0.001) (Tables 1 and 2), which is consistent with our previous reports using the same experimental approach [13, 19, 20].

### Influence of iterative reconstruction on densitometry

IR (SAFIRE) on average induced only small but significant changes in the order of ±1 HU to LD and AD compared to corresponding FBP reconstructions using medium-soft kernels (I40f and B40f, respectively) at the same exposure settings (p<0.05). For example, mean LD was -902.5 HU for FBP and -901.6 HU for IR at lowest dose (80 kV_p_ 12 mAs) (+0.1%) with high attenuating chest wall, and -926.6 HU for FBP and -925.4 HU for IR with low attenuation chest wall (p<0.05) (+0.1%) (Tables 1 and 2). Therefore, IR had far less impact on measured LD and AD than chest wall attenuation or acquisition settings. In other words, changes of LD and AD induced by changing acquisition settings, e.g. by lowering tube potential (kV_p_) and/or current-time product (mAs), are not compensated for by IR.

## Discussion

This study evaluated the influence of acquisition settings and radiation exposure in conjunction with IR on CT lung densitometry in a dedicated porcine ex vivo chest phantom. Pig lung (bronchial and lobar) anatomy is quite similar to human lung anatomy as it has a similar number of bronchial generations and also a general decrease in bronchial diameter and length seen with bifurcations. Also, for example, the left lung of pigs also has a cranial and caudal lobe, and therefore it has been previously suggested as a model for translational research in respiratory medicine by Judge et al. [27]. Specifically, we could demonstrate that lung density measured based on image data reconstructed with IR yields results comparable to FBP at different levels of radiation exposure within the range of currently used clinical protocols including low-dose acquisitions, while noise is significantly reduced by IR as expected.

As expected, mean noise in lung and air was significantly higher in the phantom mimicking high attenuation chest wall compared to low attenuation chest wall at all acquisition settings with FBP and IR, except for LD at 120 kV_p_ 120 mAs (Tables 1 and 2). To our knowledge, no study investigated different background signals in chest imaging and its consequences so far. Regarding image noise in lung and air, we were able to show that image noise increased with decreasing tube-current time product independent of the reconstruction algorithm, which is in agreement with a study of Sieren et al. [16]. This effect is also in line with a recent study of Botelho et al., who used an anthropomorphic artificial chest phantom, showing that lowering exposure parameters leads to an increase of image noise for both FBP and IR in lung lesion conspicuity [28]. Recently, Lee et al. could also show an increase of noise with reduced radiation exposure [6] for the same reconstruction algorithms (FBP/SAFIRE) within an anthropomorphic chest phantom harboring animal lungs using four different exposure levels. The impact of acquisition settings on noise in our study was also higher within the high attenuation chest wall phantom compared to the low attenuation chest wall phantom (Tables 1 and 2), which is clinically meaningful in the context of obese patients.

For our study we used a broad range of different exposure levels (eight) within a CTDIvol range of 0.25–8.07 mGy, reflecting a broad spectrum of possible chest CT exposure scenarios in an anthropomorphic ex vivo chest phantom. Sieren et al. used six different exposure levels ranging from 0.74–11.94 mGy using a foam test model with different foams representing air, lung, and emphysema density [16, 29]. They could show that lowering tube current/potential resulted in a shift of the median density values of about 1 HU with dose reduction within the groups (SAFIRE/WFBP) which is partially in agreement with our study [16]: In their study, air density increased with lower exposure settings, however, there was no significant difference in lung density [29]. This is in line with our study when looking at the high attenuation chest wall phantom where we could show very small but significant differences within varying radiation exposure in air density (Table 2) and only one significant difference result within the lung density, whereas in low attenuating chest wall phantom there were significantly different results for both lung and air density.

As initially hypothesized we could show that noise in lung and air measured for IR was significantly lower compared to FBP and reduced by approx. 31% and 28% in the high attenuation chest wall phantom respectively (22% and 26% in the low attenuation chest wall phantom), which is consistent with our previous reports using the same experimental approach [13, 19, 20]. Lee could demonstrate a noise reduction of 49% in a chest phantom at same radiation dose when comparing FBP with SAFIRE [6]. Hérin et al. made the same observations with IR from a different manufacturer (FBP vs. ASIR vs. MBIR) while using a geometrical CT (image) quality assurance phantom (Catphan 600), where there was a tendency to higher noise with FBP compared to IR and lower air density in IR [30]. As mentioned above, Sieren et al. could also show that image noise was lower with IR (SAFIRE) compared to FBP at six different exposure levels [16, 29].

IR induced only small but significant changes in the order of ±1 HU to lung density and air density compared to corresponding FBP reconstructions (Tables 1 and 2). However, the influence of IR on LD and AD was of lower magnitude than the influence of chest wall attenuation or of acquisition settings. In other words, changes of LD and AD induced by changing acquisition settings, e.g. by lowering tube potential (kV_p_) and/or current-time product (mAs), are not compensated for by IR. A recent study showed in a lung phantom model that with IR (ASIR) at same acquisition settings (4 different tube current-time products: range 12–100 mAs) the mean HU of the density standards did not differ much from FBP reconstruction (<4 HU deviation) [31].

There are some limitations of our study: Only one “strength” of IR and FBP from one single manufacturer has been used. Although absolute results are expected to differ for other types of reconstruction algorithms from other vendors, the general findings reported here can be expected to be valid nevertheless. Furthermore, we performed only one scan per dose level and phantom, and thus subtle variations in lung density due to actual variations in CT performance have not be assessed. As such variations in CT performance are to be expected over the course of several acquisitions, potential effects on lung densitometry measurements should be systematically evaluated in future work. Additionally, an anthropomorphic *ex vivo* lung phantom employing porcine heart-lung explants was used, and although featuring real lung tissue (healthy, well-inflated) results might not be fully transferable to clinical routine imaging of human beings. And also only lungs without surrounding ribs were measured, that may have had an influence on lung densitometry measurement.

Also there is a difference in anatomy, e.g. humans have a bipodial branching pattern and pigs have a monopodial branching pattern, but which is not subject to the present study and was previously investigated using a similar approach [13, 27]. This systematic limitation, however, had to be accepted as a similar study design featuring repeated CT acquisitions at several different exposure levels in patients in a clinical setting would be ethically questionable and prohibitive in view of the associated high radiation exposure resulting from repeated acquisitions. Moreover, we were not able to perform automated densitometry of the full lung [23, 32, 33] but had to resort to sampling by standardized ROIs in this ex vivo phantom, because the individual porcine lungs obviously do not perfectly fit into the fabricated phantom shell, thus leaving a small pneumothorax and pneumomediastinum surrounding parts of the lungs. For the specific geometry imposed by the anthropomorphic *ex vivo* porcine chest phantom, these render systematically reproducible and reliable automatic lung segmentation difficult, while manual segmentation on 160 datasets consisting of approx. 600 slices each does not seem feasible. Further studies are thus needed to allow for reproducible and reliable automatic approaches towards segmentation within research setups such as the anthropomorphic *ex vivo* porcine chest phantom used.

Also for further studies it could be interesting to compare noise power spectrum for FBP and IR for the selected dose levels to relate to spatial noise distribution, not only for its magnitude.

## Conclusion

In this *ex vivo* anthropomorphic phantom study using a CT system and a specific IR algorithm (SAFIRE) from a single vendor, noise increases significantly with lowered radiation exposure and higher chest wall attenuation, and IR partially mitigates effects of exposure reduction on noise, with a stronger effect for a high attenuation chest wall. While IR reduces noise, its influence on densitometry may be negligible due to a magnitude of approx. ±1 HU. IR may thus be used in the setting of densitometry, although the systematic changes of attenuation values induced by low-dose chest CT are not corrected for by use of IR. Further studies with CT systems and IR algorithms from different vendors are warranted in order to show that these results may be generalizable and transferable to clinical routine.

## Supporting information

S1 File (XLSX)
